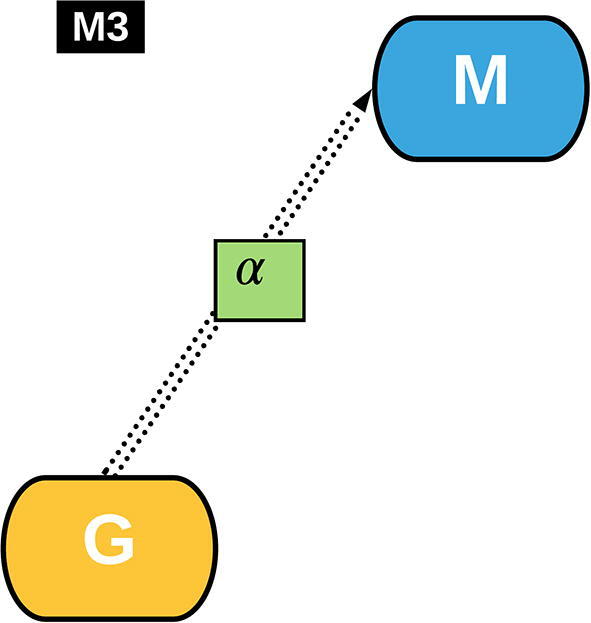# Gut microbiome mediates host genomic effects on phenotypes: a case study with fat deposition in pigs

**DOI:** 10.1016/j.csbj.2020.12.038

**Published:** 2020-12-30

**Authors:** Francesco Tiezzi, Justin Fix, Clint Schwab, Caleb Shull, Christian Maltecca

**Affiliations:** a Department of Animal Science, North Carolina State University, Raleigh, NC, USA; b Acuity Ag Solutions, LLC, Carlyle, IL 62230, USA; c The Maschhoffs, LLC, Carlyle, IL 62230, USA

**Keywords:** SNP, Single Nucleotide Polymorphism marker, OUT, Operational Taxonomic Units, SEM, Structural equation model, G, host genomic features, represented in this study by SNP, M, gut microbiome features, represented in this study by OUT, P, Phenotype recorded on the host, Π, Latent variable built on the P variables, BF1, Backfat depth measured *in vivo* at the age of 118.1±1.16 d, BF2, Backfat depth measured *in vivo* at the age of 145.9±1.53 d, BF3, Backfat depth measured *in vivo* at the age of 174.3±1.43 d, BF4, Backfat depth measured *in vivo* at the age of 196.6±8.03 d, BFt, Backfat measured *post mortem* (after slaughter at 196.6±8.03 d), BEL, Weight of the belly cut, FATg, Latent variable built on BF1, BF2, and BF3, FATt, Latent variable built on BF4, BFt, and BEL, Mod1, Model 1, used to estimate the total effect of G on P. Reported in Fig. 1a, Mod2, Model 2, used to estimate the effect of M on P. Reported in Fig. 1b, Mod3, Model 3, used to estimate the effect of G on M. Reported in Fig. S1, Mod4, Model 4, used to estimate the direct and mediated effects of G on P. Reported in Fig. 1c, Mod1L, Model 1L, used to estimate the total effect of G on, Mod2L, Model 2L, used to estimate the effect of M on, Mod4L, Model 4, used to estimate the direct and mediated effects of G on. Reported in Fig. 1d, S2a, S2b, S3a, S3b, S3c, Gut microbiome OUT selected used as mediator variables. See Table 2, Causal effect, Gut microbiome, Fat deposition, Latent variables

## Abstract

A large number of studies have highlighted the importance of gut microbiome composition in shaping fat deposition in mammals. Several studies have also highlighted how host genome controls the abundance of certain species that make up the gut microbiota. We propose a systematic approach to infer how the host genome can control the gut microbiome, which in turn contributes to the host phenotype determination. We implemented a mediation test that can be applied to measured and latent dependent variables to describe fat deposition in swine (*Sus scrofa*). In this study, we identify several host genomic features having a microbiome-mediated effects on fat deposition. This demonstrates how the host genome can affect the phenotypic trait by inducing a change in gut microbiome composition that leads to a change in the phenotype. Host genomic variants identified through our analysis are different than the ones detected in a traditional genome-wide association study. In addition, the use of latent dependent variables allows for the discovery of additional host genomic features that do not show a significant effect on the measured variables. Microbiome-mediated host genomic effects can help understand the genetic determination of fat deposition. Since their contribution to the overall genetic variance is usually not included in association studies, they can contribute to filling the missing heritability gap and provide further insights into the host genome – gut microbiome interplay. Further studies should focus on the portability of these effects to other populations as well as their preservation when pro-/pre-/anti-biotics are used (i.e. remediation).

## Graphical abstract

**Figure:**
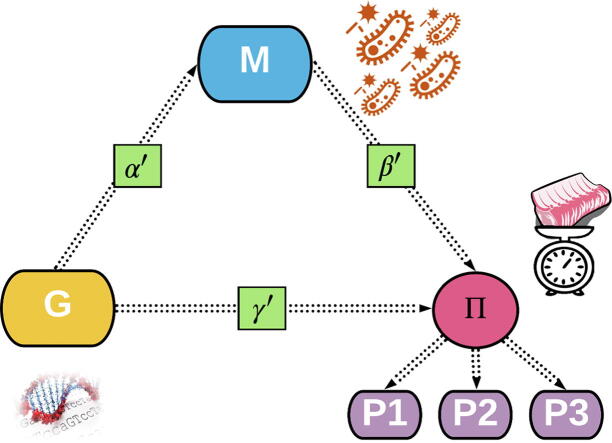

## 1 Introduction

A vast body of literature exists in both plants and livestock on discovering genomic (G) variants with a significant effect on a variety of phenotypes (P) of interest.

Recently, an increasing number of studies have started focusing on the impact of the gut microbiome (M) on phenotypic performance [54], [53], [17], where gut microbiome is expressed as the relative abundance of microbial features such as genes, taxa and biological function, etc.

In some of these studies, an additional step in which G variants are tested for their association with M features is presented [9], [64], [10]. In all these studies though, the interdependence between host genomic makeup and microbial composition is ignored. A more systematic approach to investigating the role of the host genome’s role and its microbial makeup is necessary to disentangle the complex interplay between these two components.

In the context of a genome-wide association study (GWAS), the effect of a given G feature on a P of interest is tested for its magnitude and significance. Other genetic and environmental effects are included in the (usually linear) model used to perform the association in order to adjust for effects that could be present due to the characteristics of the experimental design. An acyclic graph representation of a generic model used in GWAS (Mod1) is reported in Fig. 1a, where $\gamma_{p\leftarrow g}$ is the *total* effect of G on P. Other effects are usually included in the model to control for other design factor effects. These can be genetic g_p_ or environmental e_p_. Likewise, microbiome-wide association studies (MWAS) are performed to identify associations between M and P. A graphical representation of a generic MWAS model (Mod2) is reported in Fig. 1b, where M is the measured microbial feature and $\beta_{p\leftarrow m}$ is the effect of M on P. Again, g_p_ and e_p_ effects can be fit to account for other factor effects. Lastly, a GWAS study can be performed using M as the dependent variable of interest. In this case the generic model (Mod3) is as reported in Fig. S1, where $\alpha_{m\leftarrow g}$ is the effect of the G variant on the M feature abundance measured in each individual. Again, factor design effects can be fitted in the model as they exert an effect on M. These could, again, be genetic g_m_ or environmental e_m_.

In GWAS studies, the term *association* is emphasized in the acronym though the regression coefficient $\gamma_{p\leftarrow g}$ is normally the quantity of interest, representing the *effect* of G on P. The term *effect* carries a causal meaning, which is often neglected in the interpretation of GWAS studies. However, in this study we will refer to the estimated regression coefficients as effects, implying causality. Thus, we will refer to $\gamma_{p\leftarrow g}$ as the extent to which P changes by changing G of 1 unit [55]. We consider the G → P path in Mod1 (Fig. 1a) as single and direct and we disregard what governs the path at the biological level. In reality, a unit change in G could result in a γ change in P through a number of different biological paths. The total effect $\gamma_{p\leftarrow g}$ will then express the sum of all paths contributions to the overall effect. Let's assume that there is a *p* number of paths through which G affects P, with *p*-1 paths having null effect and one path having a non-null effect. The $\gamma_{p\leftarrow g}$ will, in this case, amount to the effect of the only non-null path, since all the null paths won’t impact the sum.

In the case of multiple paths with non-null effects but of opposite signs, the resulting $\gamma_{p\leftarrow g}$ effect will still be the sum of all paths. In this case, $\gamma_{p\leftarrow g}$ could potentially amount to zero if two or more non-null paths are of similar magnitude but opposite sign. When performing a GWAS analysis, this would result in failing to identify G features that affect P through multiple paths.

A potential solution to this problem is offered by mediation analysis [20], [35] where an intermediate variable is used as mediator between G and P. Such a variable would show an effect on P but, importantly, it would also be affected by G. Variables describing gut microbiome composition have been found to contribute to the determination of the phenotype while being affected by the host genome [48], [5]. Therefore, gut microbiome features could be used as a mediator variable for the G → P path, potentially enabling the identification of G features that could elicit a null total effect but a non-null mediated effect. A mediation model which jointly estimates the direct path G → P but also the M-mediated path G → M → P is represented in Fig. 1c. Note that the $\beta_{p\leftarrow m}$ and $\gamma_{p\leftarrow g}$ coefficients have now become ${\beta \text{'}}_{p\leftarrow m}$ and ${\gamma \text{'}}_{p\leftarrow g}$, because of their simultaneous estimation in the model. The ${\beta \text{'}}_{p\leftarrow m}$ coefficient describes the effect of M on P while holding G constant (as opposed to β) while ${\gamma \text{'}}_{p\leftarrow g}$ elicits the impact of G on P while holding M constant. For notation convenience, the $\alpha_{m\leftarrow g}$ coefficient has become ${\alpha \text{'}}_{m\leftarrow g}$, although there are no differences in the parameter interpretation. A mediation analysis allows the peeling at least one of the paths hidden in the G → P total effect, several G variants could be identified as having a M-mediated effect on P, variants that would be missed in the case of a simple GWAS. In fact, the lack of association between G and P and the null effect of G on P ($\gamma_{p\leftarrow g}$) do not imply that the G → M → P path has null magnitude. Since $\gamma_{p\leftarrow g}$ is the result of multiple paths and these could sum to zero, a single path does not necessarily equal to zero in magnitude.

Examples of such phenomenon are reported in behavioral and marketing research. For instance, Pollack et al. [39] examined the relationship between economic stress and entrepreneur’s intentions to withdraw from business. While the total effect of economic stress on withdrawing intentions was not significant, the path mediated by depressed affect showed a significant estimate. Similarly, Kampfer et al. [22] found that the packaging of food and beverages affected the willingness of consumers to purchase and consume, but this effect was mediated by the flavor intensity and evaluation of the product. In both cases, the study of the total effect alone (similarly to Mod1 in this study) could not have identified the mediated effects, estimable only through the use of mediator variables. In the field of biology, Díez-Fernández et al. [11] found that body mass index mediated the effect of muscular fitness on several cardiometabolic risk variables in children and Bliuc et al. [8] showed that a reduction in the rate of bone loss mediated the effect of nitrogen bisphosphonates on patient mortality. It is worth noting that the non-mediated effect was still present in these studies, but the mediation analysis helped in elucidating the dynamics of the total effect.

In all the models outlined above, it was implicit that the variables G, M and P were directly measured. In reality, the same models could be applied to latent, non-measured variables through Structural Equation Models (SEM) which can combine mediation analysis with latent variable analysis [43], [18]. The measured P is in this case replaced with a latent variable Π, which is built on a number of measured variables partially correlated among them [38]. Let’s for example consider a number of G features that exert a significant $\gamma_{p\leftarrow g}$ effect on two measured variables (as in Mod4). The γ effect of G on Π ($\gamma_{\Pi \leftarrow g}$) would imply an effect on the latent variable. In addition, several G features could show a significant $\gamma_{\Pi \leftarrow g}$ but not significant $\gamma_{p\leftarrow g}$ on the P components, because the latent variable can express a layer of information that lies within the measured variables but that cannot be extracted by any of them individually, typically because of limitations in *measuring* a phenotype.

The example could also apply to a G → M → Π path analysis, where M is mediator variable exerting an effect on the latent variable Π. A potential implementation for the model (Mod4L) described above is depicted in Fig. 1d, where Π is represented in a circle since it expresses a latent, non-measured variable constructed on the measured phenotypes P1, P2 and P3. The SEM allows us to test for the M−mediated effect of G on the latent variable.

A mediation analysis implies a causal process, which has to be hypothesized *a priori* and then tested on the data available data. MWAS results are usually not causally interpretable, although the need to move from association to causality has been emphasized by several authors [50], [48] along with the need to understand the role of the host organism in shaping the microbiome-phenotype link.

Swine (*Sus scrofa*) is a relevant species to study fat deposition patterns when they depend on the host genomic background [44] or the composition of the gut microbiome composition [24] together with the impact of host genomic variants on gut microbiome composition itself [10], [5]. Datasets of significant size can be easily generated using *Sus scrofa* as a model organism, kept either under experimental or commercial conditions. In this case, our group and its partners have generated a vast dataset, which contains several measures of fat deposition together with characterization of host genomic background and gut (fecal) microbiome composition.

The objectives of this study were to 1) to provide a framework for the implementation of a microbiome-mediated search of host genomic features with effect on phenotypes of interest; 2) search for host genomic variants that affect fat deposition through the modification of the gut microbiome and 3) to search for host genomic variants exerting a microbiome-mediated effect using latent variables constructed on multiple measures of fat deposition in swine.

## 2 Materials and methods

### 2.1 Data

Data used in this study came from an existing database and partial results of the research have been previously published [5]. Information was collected on a total of 1265 individuals of swine (*Sus scrofa*) raised under commercial conditions by The Maschhoffs LLC. (Carlyle, IL, USA). For each individual, several performance measures were collected, both *in vivo* and *post mortem*. Fecal microbiome samples were obtained at different time points during the trial. A total of 1,183 records with complete host genomic, host phenotypic and fecal microbiome information were available for subsequent analyses.

#### 2.1.1 Host phenotypic data

The phenotypic traits used in this study have already been described in other studies by our group [28], [30], [5]. In this study we focused on a set of traits that describe fat deposition in pigs from 4 to 7 months of life. The age span was constrained by the set up that commercial pork farms operate and encompasses pig development from weaning to puberty, which has its onset at around 5–8 months of life under commercial conditions [46], [36].

Briefly, phenotypes analyzed (**P**) included measures of backfat depth taken *in vivo* at the age of 118.1 ± 1.16 d (**BF1**); 145.9 ± 1.53 d (**BF2**) and 174.3 ± 1.43 days (**BF3**) of life, together with the day before slaughter which occurred at 196.6 ± 8.03 days (**BF4**). Measurements were taken on the right side of the pig’s back in the area corresponding to the 10th rib. These measures were collected using an ultrasound equipment (Biotronics Inc., Ames, IA, USA) at the facility where the animals were raised. An additional measure of backfat (**BFt**) was taken *post mortem* using a Fat-O-Meater system (Frontmatec A/S, Kolding, DK), again at the site of the 10th rib. Carcasses were then dissected into commercial cuts, each of which was weighted. We used the weight of the belly cut (**BEL**) for its high fat content [14], [26], which makes it suitable as an indicator of fat deposition.

The descriptive statistics as well as Pearson correlations among the selected phenotypic measures are reported in Table 1.

**Table 1:** Descriptive statistics and Pearson correlations for the phenotypic traits used (N = 1,183).

Trait^1^	Metric^2^	Mean	SD	Pearson correlations				
				BF1	BF2	BF3	BF4	BFt
BF1	cm	1.25	0.28	.				
BF2	cm	1.69	0.42	0.744	.			
BF3	cm	2.00	0.54	0.654	0.813	.		
BF4	cm	2.36	0.61	0.598	0.744	0.882	.	
BFt	cm	2.35	0.49	0.523	0.637	0.776	0.817	.
BEL	kg	18.30	2.78	0.431	0.450	0.503	0.554	0.453

^1^BF1, BF2, BF3 and BF4: measures of subcutaneous fat depth taken *in vivo* at the age of 118.1 ± 1.16 d; 145.9 ± 1.53 d, 174.3 ± 1.43 d and 196.6 ± 8.03 d, respectively. BFt: measure of subcutaneous fat depth taken *post mortem*. BEL: weight of belly cut taken *post mortem*.

^2^Cm: centimeters. Kg: kilograms.

**Table 2:** Summary of the microbial features (operational taxonomic units) used in the mediation analysis.

OTU	Stage	Genus	Descriptive statistics					Variance absorbed in M2^1^					
			Presence	Minimum	Mean	Median	Max	BF1	BF2	BF3	BF4	BFt	BEL
S2a	S2	*Peptococcus*	0.523	0	3.11	1	62	2.70	2.13	2.08	2.76	1.89	2.57
S2b	S2	*Peptococcus*	0.793	0	5.16	4	42	6.49	5.08	3.53	2.53	2.20	2.80
S3a	S3	*Peptococcus*	0.995	0	19.04	17	68	.	.	.	9.02	6.43	9.57
S3b	S3	*Butyricicoccus*	0.903	0	5.90	4	41	.	.	.	2.82	2.10	1.87
S3c	S3	*Butyricicoccus*	0.972	0	27.71	23	145	.	.	.	4.46	2.63	1.53^2^

1 Percentage of phenotypic variance absorbed. BF1, BF2, BF3 and BF4: measures of subcutaneous fat depth taken *in vivo* at the age of 118.1±1.16 d; 145.9±1.53 d, 174.3±1.43 d and 196.6±8.03 d, respectively. BFt: measure of subcutaneous fat depth taken *post mortem*. BEL: weight of belly cut taken *post mortem*.

2 Significant after FDR correction, not significant after Bonferroni correction.

#### 2.1.2 Host genomic data

All individuals were genotyped with the PorcineSNP60 v2 BeadChip (Illumina, Inc., San Diego, CA). Quality control of the markers was performed by removing single nucleotide polymorphisms (SNP) with a call rate of less than 0.99, minor allele frequency of less than 0.05 and deviation for Hardy-Weinberg equilibrium (*P* smaller than 0.001). After quality control the number of SNP remaining for further analyses was 40,542, distributed on the 18 autosomes. Genotypes were coded as the number of copies of the minor allele, i.e. 0 for AA, 1 for Aa or aA and 2 for aa, where a is the minor allele and A is the major allele. The SNP will hereinafter be referred to as host genomic features (**G**).

#### 2.1.3 Fecal microbiome data

Rectal swabs were obtained on all individuals at the ages of 18.6 ± 1.09 d (**S1**), 118.2 ± 1.18 (**S2**) and 174.3 ± 1.43 (**S3**). Fecal samples were subjected to 16S rDNA amplicon sequencing as previously described by Maltecca et al. [30]. This analysis was performed by Matatu Inc. (St. Louis, MO, USA). A total of 3001 operational taxonomic units (OTU) were generated. Counts were rarefied to 10,000 per sample. OTUs were removed if showing less than 1200 counts overall or showing a zero count in more than 80% of the samples. On the remaining variables, zero-value imputation was performed using the function *cmultrepl* from package “Zcompositions” [37] and center-log transformation was performed using the function *clr* from package “compositions” [56], both implemented in the R software [41]. The microbiome variables generated will be referred to as microbial features hereinafter (**M**).

### 2.2 Statistical analysis

#### 2.2.1 Transforming cross-classified effects into linear covariates

Since models had to be tailored to the SEM framework and the use of cross-classified effects is discouraged given the large number of solutions that need to be computed, cross-classified effects were transformed to linear covariates. This was carried out in two steps. First, a model was fitted for each phenotypic trait using the following specifications:

(1) $$P_{p}=CG+D+S+b+\varepsilon$$

where $P_{p}$ is the phenotypic record for the *p^th^* trait (centered to mean equal 0 and standard deviation equal to 1), *CG* is the fixed cross-classified effect of the contemporary group (batch of individuals of the same gender entering the same trial in the same week, 12 levels); D is the fixed covariate on the genetic line of the dam of the individual (coded as 1 vs 2); *S* is the fixed cross-classified effect of the sire of the individual (28 levels); *b* is the random effect of the batch of individuals (physical pen) where the individuals were allotted (331 levels); ε is the random residual. The choice to fit a CG effect on 12 levels that included the interaction between the batch and sex effects was due to previous analyses that showed an impact of such interaction. The number of records for each class (~90 records per class) is sufficient to estimate the effect over the 12 classes, providing more granularity in the correction.

In the second step, incidence matrices and vectors of solutions were used to define new explanatory variables as follows:

(2) $${\mathrm{CGb}}_{p}=X_{\mathrm{cg}}b_{{\mathrm{cg}}_{p}}+Z_{p}u_{b_{p}}$$

(3) $${\mathrm{Sr}}_{p}=X_{s}b_{s_{p}}$$

where ${\mathrm{CGb}}_{p}$ is the newly defined variable, which summarizes information about contemporary group and batch effects for the *p^th^* trait; $X_{\mathrm{cg}}$ is the incidence matrix for the contemporary group effect; $b_{{\mathrm{cg}}_{p}}$ is the vector of solutions for the contemporary group effect for the *p^th^* trait; $Z_{p}$ is the incidence matrix for the batch effect; $u_{b_{p}}$ is the vector of solutions for the batch effect for the *p^th^* trait; ${\mathrm{Sr}}_{p}$ is the newly defined variable for the sire of the individual for the *p^th^* trait; $X_{s}$ is the incidence matrix for the sire effect; $b_{s_{p}}$ is the vector of solutions for the sire effect for the *p^th^* trait.

The same process was applied using M as the dependent variable and generating the variables ${\mathrm{CGb}}_{m}$ and ${\mathrm{Sr}}_{m}$ for each of the *m^th^* M.

For further use, all the four newly generated variables were centered to show mean equal to 0 and standard deviation equal to 1.

#### 2.2.2 Genome-wide association study (Mod1)

The first analysis consisted of selecting the G features that were significantly associated with the phenotypic traits of interest (model Mod1, Fig. 1a). Once the variables ${\mathrm{CGb}}_{p}$ and ${\mathrm{Sr}}_{p}$ were defined, model Mod1 was fitted as:

(4) $$P_{p}=\gamma_{p\leftarrow g}G_{g}+b_{1gp}{\mathrm{CGp}}_{p}+b_{2gp}D+b_{3gp}{\mathrm{Sr}}_{p}+\varepsilon_{p}$$

where $P_{p}$, ${\mathrm{CGp}}_{p}$, *D*, ${\mathrm{Sr}}_{p}$ and $\varepsilon_{p}$ were as defined above; $\gamma_{p\leftarrow g}$ was the fixed effect of the *g^th^* G on the *p^th^* P; $b_{1gp}$, $b_{2gp}$ and $b_{3gp}$ are the regression coefficients for the three effects when regressing the *p^th^* phenotypic trait on the *g^th^* selected host genomic feature.

#### 2.2.3 Microbiome-wide association study (Mod2)

The second analysis consisted of selecting the M that were significantly associated with the phenotypic traits of interest (Mod2). The model was specified as:

(5) $$P_{p}=\beta_{p\leftarrow m}M_{m}+b_{1mp}{\mathrm{CGp}}_{p}+b_{2mp}D+b_{3mp}{\mathrm{Sr}}_{p}+\varepsilon_{p}$$

where $P_{p}$, ${\mathrm{CGp}}_{p}$, D, ${\mathrm{Sr}}_{p}$and $\varepsilon_{p}$ are defined as above; $\beta_{p\leftarrow m}$ is the effect of the *m^th^* microbial feature on the *p^th^* phenotypic trait (as in Fig. 1b); $M_{m}$ is the *m^th^* selected microbial feature; $b_{1mp}$, $b_{2mp}$ and $b_{3mp}$ are the regression coefficients on the effects of contemporary group (combined with batch), dam line and sire as generated when regressing the *p^th^* phenotypic trait on the *m^th^* selected microbial feature.

#### 2.2.4 Mediation path analysis (Mod4)

Model Mod4 (Fig. 1c) was used to test for the mediated path of G on P trough M [39], [20]. The estimation of the coefficients was performed simultaneously using the following specifications:

(6) $$\left\{\begin{array}{c} M_{m}={\alpha \text{'}}_{m\leftarrow g}G_{g}+b_{1gm}{\mathrm{CGb}}_{m}+b_{2gm}D+b_{3gm}{\mathrm{Sr}}_{m}+\varepsilon_{m}\\ P_{p}={\beta \text{'}}_{p\leftarrow m}M_{m}+{{\gamma \text{'}}_{p\leftarrow g}G_{g}+b}_{1gmp}{\mathrm{CGb}}_{p}+b_{2gmp}D+b_{3gmp}{\mathrm{Sr}}_{p}+\varepsilon_{p} \end{array}\right)$$

where $P_{p}$ is the *p^th^* P, $M_{m}$ is the *m^th^* M, ${\alpha \text{'}}_{m\leftarrow g}$ is the effect of the *g^th^* G on the *m^th^* M, ${\beta \text{'}}_{p\leftarrow m}$ is the effect of the *m^th^* M on the *p^th^* P, ${\gamma \text{'}}_{p\leftarrow g}$ is the effect of the *g^th^* G on the *p^th^* P; $b_{1gm}$, $b_{2gm}$ and $b_{3gm}$ are the regression coefficients for the three effects on the *m^th^* M; $b_{1gmp}$, $b_{2gmp}$ and $b_{3gmp}$ are the regression coefficients for the three effects on the *p^th^* P; D, ${\mathrm{CGb}}_{p}$, ${\mathrm{Sr}}_{p}$, ${\mathrm{CGb}}_{m}$ and ${\mathrm{Sr}}_{m}$ are as defined above; $\varepsilon_{p}$ and $\varepsilon_{m}$ are the residual errors for the *p^th^* P and *m^th^* M, respectively. This model allows to estimate the effect of G on both M and P. While estimating such effects the other effects are simultaneously estimated, in this case, the effects of CG, dam line and pen for their effect on both M and P.

#### 2.2.5 Mediation analysis in the structural equation model (Mod4L)

Measured variables contain information that is limited by the equipment and time of recording as well as the inability of the measuring technology to describe the whole biological process that is manifested in the phenotype. A SEM can be used to test the mediation through M on latent variables, providing further insights on the mediation paths provided that the latent variables built on correlated variables recover more information than the sum of the information contained in the measured variables. Model Mod4L (Fig. 1d) was implemented for this purpose.

We defined a first latent variable (**FATg**) describing the (back) fat deposition over the life of the individual using the variables BF1, BF2 and BF3. A second latent variable (**FATt**) was defined using the measured variables BF4, BFt and BEL. This second variable aimed at describing the fat deposition at the end of the trial, merging information measured on the back of the body by two different instruments (BF4 and BFt) to the weight of the cut with the largest fat content (BEL). The Pearson correlations among the six variables are reported in Table 1. While being taken at approximately the same time, the mechanical assessment of fat depth (BFt) showed a reduced variability as compared to the ultrasound measure (BF4) and a non-complete overlap (correlation less than unity). This could be due to the impact of slaughter or an actual lack of precision of the mechanical instrument. Regardless, the difference between the measures warrants the necessity of using latent variable.

The structural equation model Mod4L was defined as:

(7) $$\left\{\begin{array}{c} M_{m}={\alpha \text{'}}_{m\leftarrow g}G_{g}+b_{1gm}{\mathrm{CGp}}_{m}+b_{2gm}D+b_{3gm}{\mathrm{Sr}}_{m}+\varepsilon_{m}\\ \Pi_{\pi }={\beta \text{'}}_{\pi \leftarrow m}M_{m}+{{\gamma \text{'}}_{\pi \leftarrow g}G_{g}+b}_{1gm\pi }{\mathrm{CGp}}_{\pi }+b_{2gm\pi }D+b_{3gm\pi }{\mathrm{Sr}}_{\pi }+\varepsilon_{\pi } \end{array}\right)$$

where ${\alpha \text{'}}_{m\leftarrow g}$, $G_{g}$, $M_{m}$, $b_{1gm}$, $b_{2gm}$, $b_{3gm}$, ${\mathrm{CGp}}_{m}$, D, ${\mathrm{Sr}}_{m}$, $\varepsilon_{m}$ and $\varepsilon_{\pi }$ are as defined above; $\Pi_{\pi }$ is one of the two latent variables (FATg or FATt); ${\beta \text{'}}_{\pi \leftarrow m}$ and ${\gamma \text{'}}_{\pi \leftarrow g}$ are the effects of the *m^th^* M and *g^th^* G on the latent variable $\Pi_{\pi }$; $b_{1gm\pi }$, $b_{2gm\pi }$ and $b_{3gm\pi }$ are the regression coefficients of the latent variable $\Pi_{\pi }$ on the three design variables; ${\mathrm{CGp}}_{\pi }$ is a latent variable built upon the CGp for each of the three phenotypic traits that make the latent variable $\Pi_{\pi }$, similarly to ${\mathrm{Sr}}_{\pi }$ that is built upon the three *Sr* variables. The two variables (${\mathrm{CGp}}_{\pi }$ and ${\mathrm{Sr}}_{\pi }$) were latent variables used to adjust the latent variable $\Pi_{\pi }$ for the design effects of contemporary group and sire, respectively. These also were defined as latent variables were, with loadings estimated when running the model.

#### 2.2.6 Additional models (Mod1L, Mod2L)

In order to perform a GWAS and MWAS on the latent variables $\Pi_{\pi }$, models in [4], [5] were modified to account for the latent variables as dependent variables. Briefly, the effects of the G features were calculated using model **Mod1L**:

(8) $$\Pi_{\pi }={{\gamma \text{'}}_{\pi \leftarrow g}G_{g}+b}_{1g\pi }{\mathrm{CGp}}_{\pi }+b_{2g\pi }D+b_{3g\pi }{\mathrm{Sr}}_{\pi }+\varepsilon_{\pi }$$

And the effects of the M features were calculated using model **Mod2L**:

[9] $$\Pi_{\pi }={\beta \text{'}}_{\pi \leftarrow m}M_{m}{+b}_{1m\pi }{\mathrm{CGp}}_{\pi }+b_{2m\pi }D+b_{3m\pi }{\mathrm{Sr}}_{\pi }+\varepsilon_{\pi }$$

#### 2.2.7 Model implementation

The model in equation 1 was implemented in the R package *lme4* [1], all the other models were implemented using the R function *lavaan* [45]. First, models Mod1 and Mod1L (formulas in 4 and 8) were implemented to perform a GWAS and calculate G total effects on both measured and latent variables. The second step implemented model Mod2 to select the M features that would simultaneously affect the 3 measured variables that make up each latent variable. This selection was performed under the assumption that only the M features generating a strong β estimate could produce a significant mediated effect. In practice, we considered the M features with a Bonferroni adjusted significance (R function *p.adjust* from native package “stats”, [41] of the $\beta_{p\leftarrow m}$ effect over BF1, BF2 and BF3 for modeling FATg. Similar significance criteria were applied to BF4, BFt and BEL for FATt. For each of the tested M features, the variance absorbed by the effect was calculated as the variance of the vector generated as $\beta_{p\leftarrow m}M_{m}$. Only the M features that consistently absorbed more than 1% of the variance of $P_{p}$ were then considered. This special selection was carried out for an efficient implementation of the latent variable analysis in the structural equation model. For additional information on the impact of the M features on the traits of interest, see Bergamaschi et al. [6]. Following this step, the M features (Table 2) were identified. While features recorded at S2 were tested on all measured and latent variables, features recorded at S3 were only used on traits BF4, BFt, BEL and FATt given the incompatibility with the causal assumption that M features sampled at S3 could affect a phenotype measured at time S2.

With the selected M features, models Mod4 and Mod4L (formulas in equations 6 and 7 were selected to generate estimates of α', β' and γ' coefficients for each of the 40,542 G features.

Once the mediation path model was fitted, indirect effects were calculated as ${\alpha \text{'}}_{m\leftarrow g}\times {\beta \text{'}}_{p\leftarrow m}$ and ${\alpha \text{'}}_{m\leftarrow g}\times {\beta \text{'}}_{\Pi \leftarrow m}$ for measured and latent variables, respectively.

#### 2.2.8 Determining significance

Significance of the fitted effects in models Mod1, Mod4 and M5 (namely the effects α', β', γ' and γ) was obtained deterministically by testing the estimated probability of the parameter to be equal to zero. Significance of the $\alpha^{\text{'}}\times \beta \text{'}$ mediation path was obtained in a deterministic fashion via the Sobel test [49]. However, the Sobel test relies on the assumption of normality of the sampling distribution for the $\alpha^{\text{'}}\times \beta \text{'}$ product, which is likely inappropriate [19]. Thus, we used two independent empirical tests to evaluate the significance of the mediation path. First, a bootstrapping procedure was followed as proposed in Hayes [20] and Pollack et al. [39]. Here, N records were sampled, with replacement, for each round of bootstrapping in order to obtain an empirical distribution of parameter estimates around their point estimate obtained from the whole dataset. The process was repeated for 1000 rounds. Empirical confidence intervals at 95% probability were then calculated and significance (for *P* smaller than 0.05) was declared if the value 0 did not fall within the confidence intervals, i.e. the empirical distribution did not include null values. A permutation test was specifically designed to disrupt the G→M→P indirect path, in favor of the G→P direct path. Elements in the M column were shuffled to obtain estimates of α' and β'. Once the indirect path is broken by altering the link between and G and M and M and P, we can assume that the estimates of α' and β' under permutation do not deviate from the null hypothesis. Permutation and estimation was repeated 1000 times, then empirical confidence intervals at 95% probability were calculated on the permuted estimates and significance was declared (for *P* smaller than 0.05) if the non-permuted estimate did not fall within the confidence intervals, i.e. the null-hypothesis distribution did not include the estimate values from the intact data.

The two tests attempt to account for multiple path complexity in different ways. The permutation test aims at breaking the mediated path specifically, providing a null-hypothesis distribution for the estimates of that specific path. The bootstrapping test aims at generating a distribution of the parameter estimates given the data.

The two tests were run for models Mod4 and Mod4L only applied to selected G-M−P combinations. For each G-M−P run, the ratio $(\alpha^{\text{'}}\times \beta^{\text{'}})/\gamma \text{'}$was calculated and only combinations for which the value was larger than *1* were selected for empirical testing. This was done to select G-M−P combinations for which the mediated path was stronger than the direct path. Models Mod1 and Mod1L were instead tested only with bootstrapping and when showing a *P*-value for the γ effect below 0.01. This allowed us to generate empirical significance values for all the coefficients.

### 2.3 Comparison with previously discovered QTL in swine.

The analysis performed to discover the G variants exerting a significant total or mediated effects gave a list of markers that were compared to the existing Animal QTL database [21], www.animalgenome.org, release 41). First, markers that fell within a contiguous window of 10 SNP were grouped together. The whole *Sus scrofa* QTL database was downloaded and previously discovered QTL were merged to each of the marker windows of interest. Then, the count for each QTL category (*Exterior*, *Health*, *Meat and Carcass*, *Production*, *Reproduction*) was calculated for each of the analyses conducted (separately for total and mediated effects).

In addition, the R package *biomaRt* [15], [16] was used to retrieve candidate genes overlapping with the genomic regions identified, based on the current gene annotations from the ENSEMBL Genes platform (Version 99; www.ensembl.org/index.html).

## 3 Results

### 3.1 Mediator variables

Table 2 reports the five M features that were used as mediator variables in this study. Two of the chosen variables were recorded at S2, simultaneously to the recording of BF1, BF2 and BF3. These variables (S2a and S2b) consisted of OTU assigned to the Genus *Peptococcus* but showed a different rate of presence (frequency of non-zero counts). S2a showed a presence of 0.523 and absorbed 2.70, 2.13 and 2.08 percent of phenotypic variance for BF1, BF2 and BF3, respectively. S2b showed a stronger presence of 0.793 and absorbed 6.49, 5.08 and 3.53 percent of total variance for the three phenotypic traits.

The other three chosen M features belonged to samples collected at S3, contemporary with the recording of BF4, BFt and BEL. These M features were not tested on BF1, BF2 and BF3 for an evident conflict with the causal M → P structure with P appearing before M could be recorded. The variables S3a belonged to the same OTU as S2a and therefore to the genus *Peptococcus*, while S3b and S3c belonged to the genus *Butyricicoccus*. S3a showed stronger presence compared to S2a (0.995 vs 0.523) meaning that the relative abundance of this OTU increases as pigs age. S3a absorbed 9.02, 6.43 and 9.57 percent of the total variance for BF4, BFt and BEL, respectively, being the M feature with the strongest impact on the studied traits. S3b and S3c showed a similar presence (0.903 and 0.972) and absorbed 2.82, 2.10 and 1.87 percent of the total variance of BF4, BFt and BEL, while S3c absorbed the 4.46, 2.63 and 1.53 percent of the variance of the three P traits. It should be noted that S3c did not show a significant effect on BEL after Bonferroni correction but the effect was still significant after FDR correction. This M feature was still chosen as a mediator for the large percentage of variance absorbed on the three variables.

### 3.2 Total effect on measured and latent variables

Tables 3 and S1 show the number of G features discovered as significant for the G → P total effect, assuming no mediation (model Mod1, Fig. 1).

The number of features identified as significant after bootstrapping varied across the traits, ranging from 237 for BF1 to 497 for BFt, with intermediate values of 314 for BF2, 273 for BF3, 482 for BF4, and 265 for BEL. The latent variables identified a different number of significant G features as compared to the measured component traits. Fig. 2a and Table S2 report the number of G features that were identified as significant for the latent variable and also for the measured variables. A total of 495 features were significant for FATg (Table 3). Of these, 126 were also significant for BF1, 249 were also significant for BF2 and 119 were also significant for BF3, whereas 203 of those features were not found to be significant for any of the three measured P variables. A total of 358 G features were significant for FATt (Table 3). Of these, 202 were also significant for BF4, 216 were also significant for BF and 44 were also significant for BEL, whereas 77 were not significant for any of the measured P variables. At the same time, 65 features were found significant only for BF1, 48 for BF2, 101 for BF3, 260 for BF4, 44 for BFt and 128 for BEL (Fig. 2a).

**Fig. 1ab:**
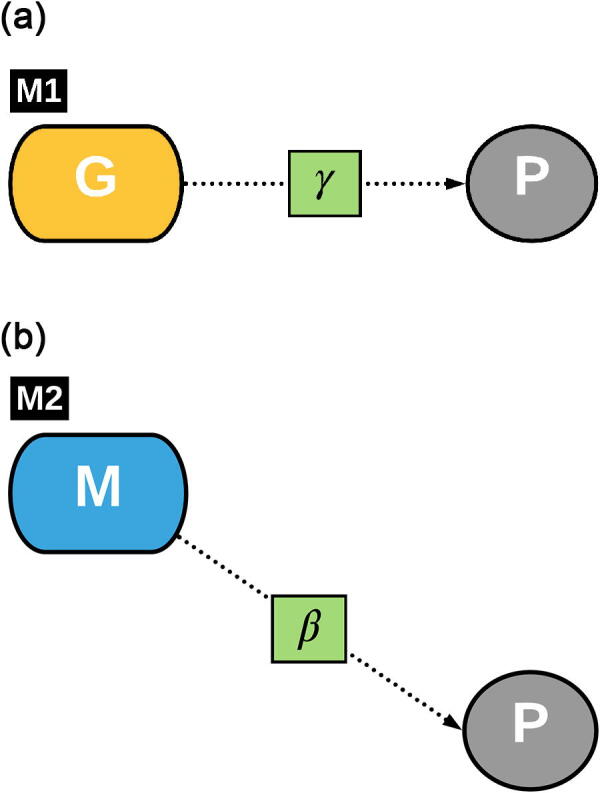
Graphical representations of the first two models used in this study. Model M1 estimates the *total* effect $\gamma_{p\leftarrow g}$ of G (host genomic features) on P (host phenotypes). Model M2 estimates the $\beta_{p\leftarrow m}$ effect of M (gut microbial features) on P (host phenotypes).

**Fig. 1cd:**
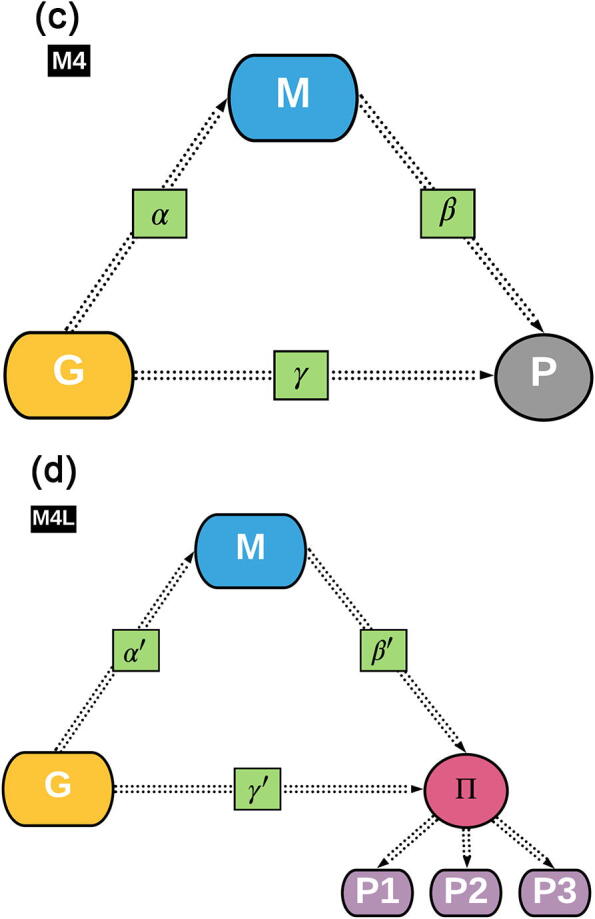
Graphical representations of the first two models used in this study. Model M4 estimates the *direct* effect ${\gamma \text{'}}_{p\leftarrow g}$ that G (host genomic features) exerts on P (host phenotypes) as well as the ${\alpha \text{'}}_{m\leftarrow g}\times {\beta \text{'}}_{p\leftarrow m}$ effect of G on P as *mediated* by M (gut microbiome). Model M4L replaces the measures P phenotype with a latent variable Π constructed on three measured phenotypes (P1, P2, P3).

**Fig. 2:**
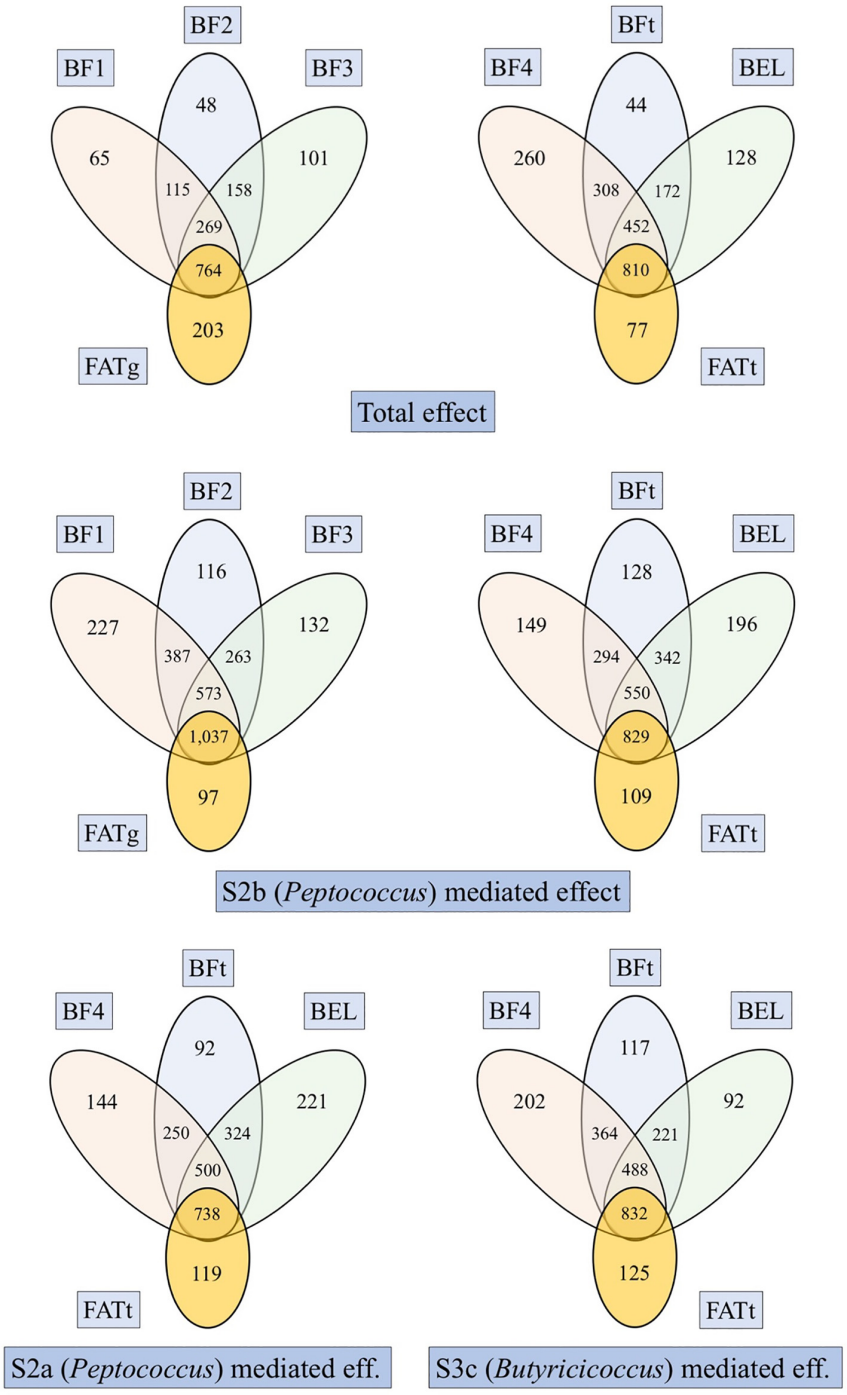
Venn diagram reporting the number of host genomic features that were declared significant for the empirical tests. Total effects (2a), mediated effects (2b, 2c).

**Table 3:** Summary of results for the M1, M1L, M4 and M4L models.

Trait^1^	Total^2^	OTU^3^	Indirect^4^	
			Mediation	Emp. Test
FATg	495	S2a	1069	0
		S2b	2311	464

BF1	237	S2a	1326	225
		S2b	2571	492

BF2	314	S2a	1255	230
		S2b	2337	461

BF3	273	S2a	1230	189
		S2b	1874	333

FATt	358	S2a	1240	238
		S2b	1611	279
		S3a	2971	0
		S3b	1420	1
		S3c	1707	344

BF4	482	S2a	1139	212
		S2b	1411	264
		S3a	2896	659
		S3b	1470	243
		S3c	1868	380

BFt	497	S2a	1136	202
		S2b	1580	299
		S3a	2383	526
		S3b	1217	207
		S3c	1607	292

BEL	265	S2a	1454	292
		S2b	1679	308
		S3a	3114	747
		S3b	1338	220
		S3c	1180	176

1 Measured (host phenotypic) or latent variables used in the analysis.

2 Number of host genomic features identified as significant for the total effect (G → P, models M1 or M1L) using bootstrapping.

3 Gut microbial feature used as mediator for the estimation of the indirect effect (G → M → P, models M4 or M4L).

4 Number of host genomic features identified as significant for the indirect effect (G → M → P, models M4 or M4L) using the Bootstrapping and Permutation tests.

### 3.3 Mediation analysis for measured variables

Table 3 also reports the number of G features with a value of the mediation ratio $(\alpha^{\text{'}}\times \beta^{\text{'}})/\gamma \text{'}$ larger than 1. It also shows how many were also found to be significant for the G → M → P indirect mediated effect after bootstrapping, assuming mediation occurs through the M features reported on the third column. Again, the number of discoveries varied greatly depending on the P but also the M variables. Fewer G features were identified for S2a as compared to the S2b mediators. Specifically, there were 492, 461 and 333 G features with S2b-mediated effects on BF1, BF2 and BF3, respectively, whereas there were 225, 230 and 189 S2a-mediated G features identified for the same traits. All the identified G features passed both the bootstrapping and the permutation tests, although the former appeared to be more conservative than the latter (see Table S1 for details). There were 212, 202 and 292 S2a-mediated G features identified for BF4, BFt and BEL, respectively, whereas there were 264, 299 and 308 S2b-mediated features identified for the same traits. The M features S3a provided, in absolute, the largest number of discoveries on the measured variables, with 659, 526 and 747 variables for BF4, BFt and BEL, respectively. S3b and S3c showed values similar to S2a and S2b, with 243, 207 and 220 discoveries for S3b and 380, 292 and 176 discoveries for S3c on the same P measured variables.

### 3.4 Mediation analysis for latent variables

Results for the mediation analysis are presented in Tables 3 and S1. The number of discoveries for latent variables was substantially different that the discoveries for each of the component measured variables. FATg was found to be affected by 464 S2b-mediated G features, while none of them passed both the empirical tests for significance when S2a was used. Similarly, only one S3b-mediated G features was found significant for FATt and none were identified when S3a was used as a mediator. In contrast, 238 features were found to be S2a-mediated, 279 were S2b-mediated and 344 were S3c-mediated. It should be noted that the permutation test appeared to be more conservative than the bootstrapping test when few or no G features were identified for their mediated effect on either FATg or FATt.

**Fig. 3:**
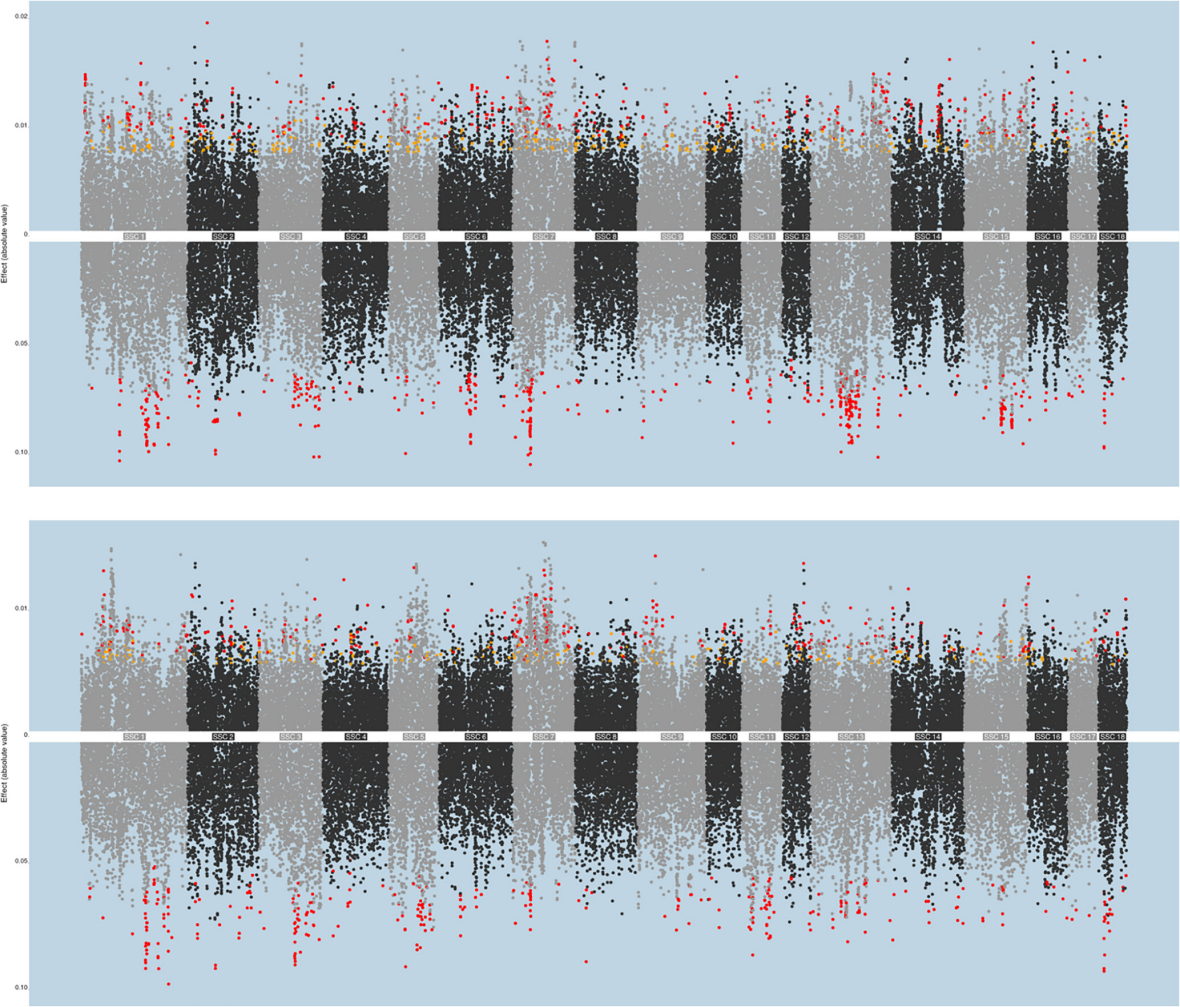
Miami plot reporting the mediated (above) and total (below) effects (as absolute values) estimated on FATg (3a) and FATt (3b). Yellow dots represent features that were selected for empirical test but did not result significant. Red dots represent features that resulted significant. (For interpretation of the references to colour in this figure legend, the reader is referred to the web version of this article.)

Fig. 3 reports Miami plots for the total and mediated effects of the G features on the two latent variables (Fig. 1a for FATg, Fig. 1b for FATt).

The mediator variable used is the one that allowed the largest number of discoveries, namely S2b for FATg and S3c for FATt (Table 3). In the plots, the upper part shows the magnitude of the mediated effect (absolute value of the $\alpha^{\text{'}}\times \beta^{\text{'}}$ product) while the lower part reports the magnitude of the total effect (absolute value of the γ effect). Dots are colored in red if they passed the empirical tests or in yellow if they were selected for the mediation test but did not pass the empirical tests. Both plots show that the genomic regions identified for the total or mediated effects spanned across all chromosomes with no overlapping between the two effects. For FATg, the mediated effect was mostly mapped to the SSC 7 (134,388,813 bp, 91,966,976 bp, 95,549,575 bp), 2 (30,870,402 to 30,999,341 bp), 10 (66,364,425 bp), 6 (147,237,661 bp), 16 (7,358,991 bp, 9,664,975 to 9,811,863 bp, 2,931,783 bp), 5 (110,792,250 bp), 4 (12,603,508 bp), 8 (134,362,111 bp), 13 (205,628,626 to 205,687,638 bp), 17 (37,920,822 to 37,938,790 bp), while the total effect was mapped to SSC 3 (126,974,179 bp), 5 (28,604,085 bp), 2 (59,957,369 to 60,318,677 bp), 1 (219,816,097 to 221,276,670 bp, 193,610,978 to 194,258,004 bp, 74,467,285 to 75,058,189 bp, 74,467,285 to 75,058,189 bp, 183,336,668 to 185,654,681 bp, 189,896,026 bp), 9 (8,014,440 bp), 7 (4,076,029 to 4,095,479 bp), 15 (118,809,721 bp), 13 (95,481,059 to 99,700,583 bp, 77,535,708 to 77,558,751 bp), 6 (63,310,472 to 63,895,672 bp), 18 (9,598,458 to 9,979,869 bp). Even in SSC 2 and 7, the peaks for the two effects did not overlap. Also for FATt, genomic regions of interest could be found on several SSC. Specifically, regions of interest for the mediated effect were found on SSC 9 (26,759,246 bp), 5 (63,702,753 bp), 4 (34,924,383 to 35,367,430 bp), 12 (various markers between 47,346,977 and 47,838,946 bp), 16 (508,634 to 1,521,611 bp), 7 (65,904,152 bp, 62,228,449 to 62,443,707, 90,198,337 to 90,335,338 bp and various markers from 4,5015,518 to 45,338,962 bp and from 27,389,874 to 27,895,570 bp), 1 (33,766,865 to 34,359,698 bp), 2 (5,883,882 to 5,885,864 bp, 5,886,990 to 5,906,388 bp and 7,261,756 to 7,282,608 bp), 14 (26,641,666) and 3 (129,063,098) while the total effect was mapped to SSC 1 (242,984,908 bp, 193,610,978 to 194,258,004 bp, 240,661,253 to 241,120,799 bp, 284,208,765 bp), 2 (59,957,369 to 60,261,433 bp, 132,702,219 bp), 5 (28,604,085 bp, 68,326,348 to 68,505,852 bp), 11 (20,953,383 bp, 68,288,395 to 68,312,256 bp), 3 (103,288,341 bp, 83,365,231 to 87,200,902 bp, 135,861,845 bp), 14 (1,000,181 bp) and 18 (9,598,458 to 9,979,869 bp). While the regions on SSC1 were different between the two effects, in this case there was a closer proximity between the significant regions on SSC5, yet the regions were not overlapping.

Fig. 2b and c (and Table S3) show the number of G features that were identified as significant for the mediated effect on the latent variables and on any of the measured variables, or none. The cases of S2a-mediated effects on FATg and S3a/S3b-mediated effects on FATt will not be listed due to the lack of discovery on the latent variables. In the case of S2b-mediated discovery on FATg, 97 were not identified on measured traits while 227, 116 and 132 were only identified on BF1, BF2 and B3, respectively. In the case of S2b-mediated discovery on FATt, 109 were not identified on measured traits while 149, 128 and 196 were only identified on BF4, BFt and BEL, respectively. Similar figures were found on FATt for the S2a and S3c-mediated effects, with 119 and 125 G features only identified on the latent variables, whereas 144, 92 and 221 features and 202, 117, and 92 features identified also on BF4, BFt and BEL.

### 3.5 Comparison with previously discovered QTL in swine

**Fig. 4:**
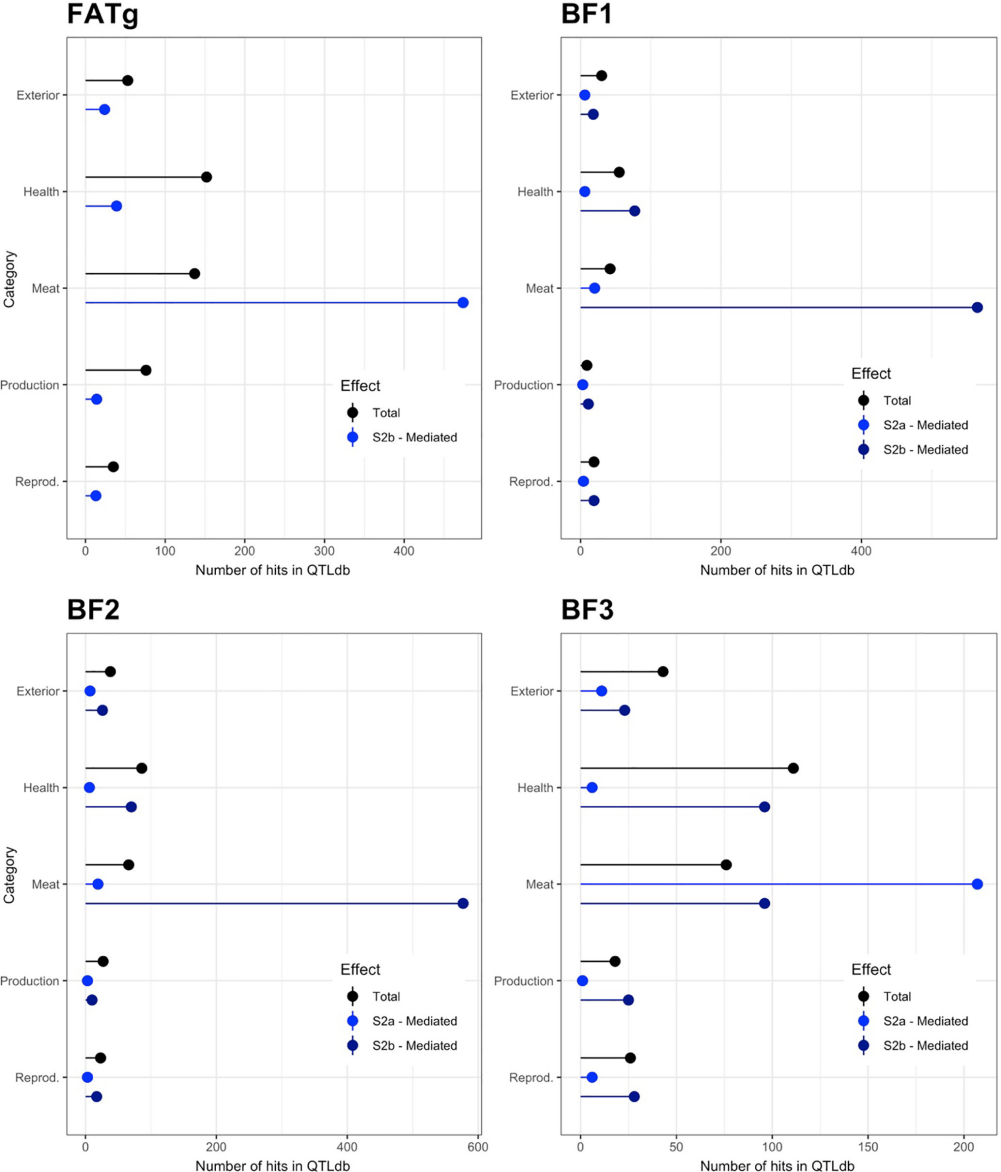

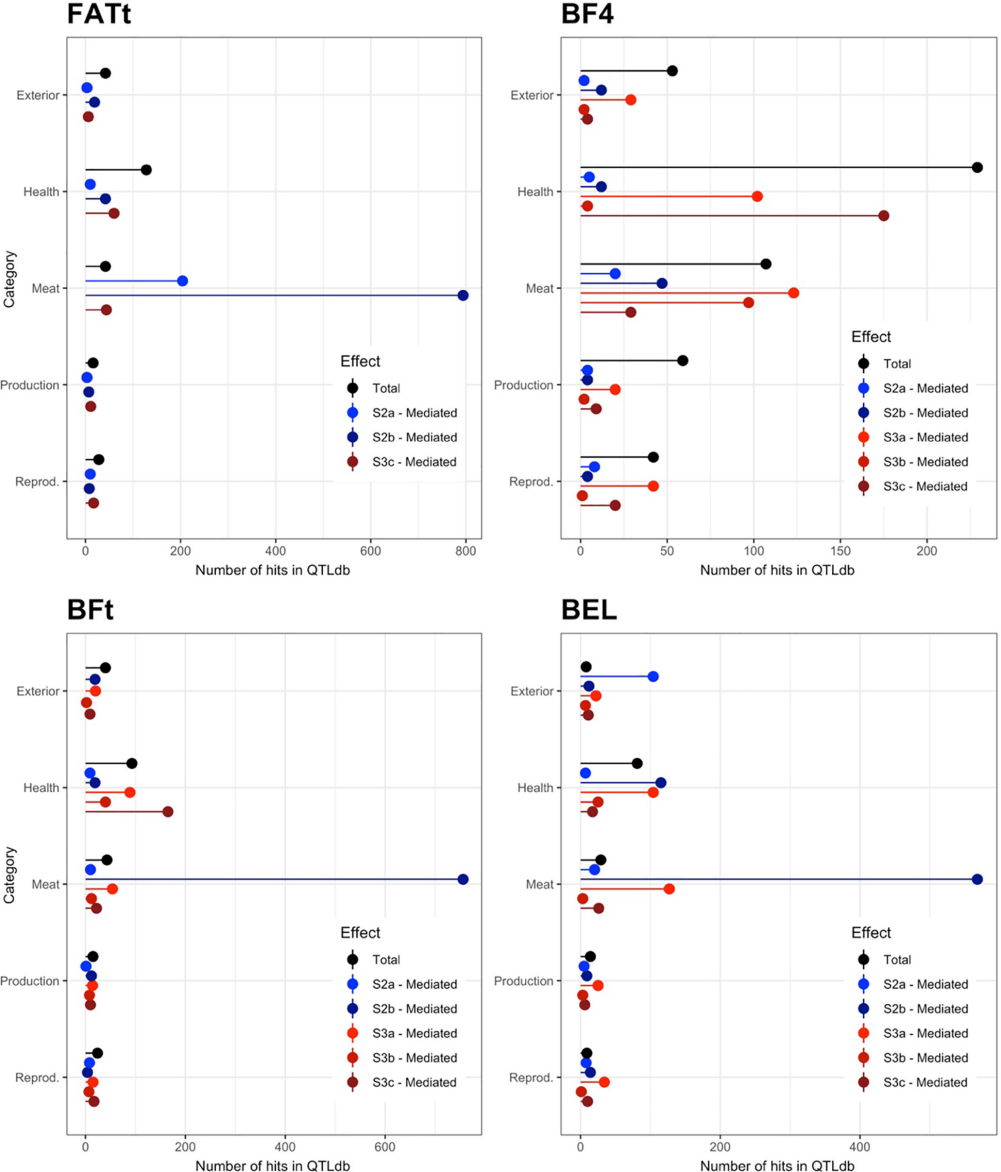
Plots reporting the number of host genomic features that were declared significant for the empirical tests as assigned to the different categories of QTL previously reported in the *Sus scrofa* literature (www.animalgenome.org). Fat growth (4a) and fat terminal (4b) traits.

Fig. 4 shows the number of QTL reported in QTLdb [21]; www.animalgenome.org) that were found in proximity to the G features discovered in this study (hereinafter called ‘QTL hits’). The results for the Total effect are equivalent to the results that can be obtained with a traditional GWAS.

In the online database, the QTL are grouped based on the trait category they were found in association to. These categories are reported on the y-axis, while the number of QTL hits is reported on the x-axis. According to the database categories, the six traits used in this study belong to the *Meat and Carcass Traits* category, here referred to as *Meat*.

With the exception of BF4, the largest number of hits per trait was reached by a mediated effect on the *Meat* category. S2a-mediated effects were the largest for BF3 and FATg while S2b (*Peptococcus*) mediated effects showed the largest number of hits for BF1, BF2, BFt, BEL and the latent variable FATt. The largest number of hits in the *Meat* category was always reached by one of the mediated effects. For BF4, the total effect showed a larger number of hits in the *Health* group, followed by the S3c-mediated effect in the same group. Yet, there was a consistent number of hits in the *Meat* group, with ~100 hits from the total effect and the S3a-/s3b-mediated effects.

## 4 Discussion

### 4.1 Mediation analysis allows the discovery of relevant host genomic variants

This study discovered several host genomic variants that affect fat deposition in swine involving gut microbiome as a mediator. In the analysis implemented, the effect that the host genome exerts on the phenotypic traits of interest is modeled as a *direct* effect but also as an effect on the microbiome features, which in turn affect the phenotypic trait of interest. While the direct effect assumes that a change in the host genome causes a change in the phenotypes with no assumption on the biological path that could lead to that change, the mediated effect assumes that the mediator (the gut microbiome composition) is involved in the path to test. The mediation analysis, therefore, compares a specific biological path (the mediated effect) to a generic path (the direct effect). Because of the nature of this study, our discussion does not deeply focus on the results about the G and M features discoveries, but on how mediation analysis implemented in a SEM can help recover host genomic variance for fat deposition traits.

Several hypotheses on the biological mechanisms involved in mediated effects are possible. For example, some G features could alter the microbiota composition (G → M), either by providing a more or less conducive substrate for a particular group of bacteria. That alteration could in turn result in a cascade of alterations leading to a different fat deposition pattern (G → M). The reason why this biological path could not be observed (and estimated) with a traditional GWAS (G → M) is because additional mechanisms interfere with the changes occurring in the mediated path. Such mechanisms could be normally occurring molecular processes aimed at maintaining a similar fat deposition homeorhesis despite of the variability in G. For example, such additional mechanism could involve the sense of satiety and lead to a change in feeding behavior, which in turn would reduce fat accumulation.

It should be further noticed that fecal samples do not represent the microbiota composition of the different intestinal tract. The use of caecal samples as opposed to rectal swabs could provide a more granular description of the intestinal microbiota composition thus improve the model performance and the understanding of the complex dynamics among the host and the microbiota [29].

Several phenotypic traits were used in this study to describe fat deposition of pigs raised in commercial conditions for meat production. These traits were grouped in order to describe fat deposition across the pig’s growth period. The first step was to select gut microbial features that would show a consistent impact on the three traits that would compose each group. The OTU that were used for this purpose belong exclusively to the *Peptococcus* and *Butyricicoccus* genera, which showed different presence and abundance during the two stages of sampling yet exerted a strong impact on the three traits of each group. These genera have already been found to be differently abundant in pig with high and low feed conversion ratio by Tan et al. [51]. *Peptococcus* was also found to be positively associated with fat deposition in a study conducted by our group in a separate cohort of pigs [5].

*Butyricicoccus* was found to be negatively correlated with a number of clinical indicators by Zeng et al. [63] and, unsurprisingly, was also discovered as a mediator of probiotic and antibiotic administration aimed at reducing food allergies in mice [58].

The mediation analysis was compared to a traditional GWAS (Mod1, Fig. 1), estimating the *total* effect that the host genomic variants exert on the phenotype regardless of the biological path taken. The mediation analysis allowed the discovery of a number of features that were equal in number, if not more, than those discovered for the *total* effect (Table 3).

While the number of significant associations that can be discovered as significant in GWAS depends mostly on the size of the experimental design and the heritability of the trait, we noted variability in the number of features identified in the mediation analysis. This variability appeared to depend on the mediator variable used with OTU assigned to the genus *Peptococcus* allowing more discoveries than the others (S2b and S3a for FATg and FATt, respectively). These two mediator variables were also the ones with the strongest and most consistent effects on the phenotypic traits (Table 2), expressed as the ${\beta \text{'}}_{p\leftarrow m}$ effect in the MWAS model (Mod2, Fig. 1). Since a strong mediated effect is built on the product of the ${\alpha \text{'}}_{m\leftarrow g}$ and ${\beta \text{'}}_{p\leftarrow m}$ effects, both effects being strong to results in a significant indirect effect estimation. Therefore, we could infer that an essential step for building a robust microbiome-mediated host genome scan is to employ mediator variables with a strong effect on the phenotypic trait. Mediators that show a weak ${\beta \text{'}}_{p\leftarrow m}$ effect could still allow strong mediated effects, but further research is needed on how to identify them in using a time-efficient method.

### 4.2 SEM allows the extraction of latent information

Structural equation modeling and the use of latent variables allow the extraction of information that cannot be recovered by single traits. In this study, the fat deposition traits were grouped to describe adipose tissue deposition at different stages of the growth period and in different parts of the body at the end of the study. In the former of the two, the use of multiple variables is advisable because growth trajectories differ between individuals for different management and physiological factors [32]. As with whole-body growth, fat deposition can take different patterns. In the latter of the two cases, multi-instrument multi-tissue measures of fat deposition were used in order to extract information on fat deposition that goes beyond what can be observed in a single part of the body.

When the latent variables were used as the dependent and most endogenous variables, several host genomic variants were identified showing a *mediated* effect larger than the *direct* one. In some cases, no G features passed the empirical significance tests although in others novel G features were identified. For example, the M feature S2b (*Peptococcus*) allowed the overall discovery of 464 features for FATg and 279 for FATt (Table 3). Of these, 97 and 109 could only be identified by the latent variables (Fig. 2b) using model Mod4L.

### 4.3 Total and mediated effects are mapped to different genomic regions

The magnitudes of the mediated G → M → P (above) and total G → P (below) effects are depicted in Fig. 3 for scenarios where most of the G features could be identified through a mediated effect. Mediated effects were in this case smaller, which is probably due to the fact that two specific phenomena have to occur for the mediated effect to be detected, while multiple paths could be involved in the total effect.

The host genomic regions that are responsible for the *total* and *mediated* effects were generally different. Fig. 4 reports a description of the concordance between the G features identified in this study and the QTL reported for *Sus scrofa* [21]; www.animalgenome.org). There was seldom an agreement between the total and mediated effects for their allocation on the QTL categories since the number of hits for the two effects was different for most of the traits. The total effects were predominantly associated with *Health* and *Meat* QTL. The fact that the *Health* category showed several hits for the total effect could be due to the fact that the animals used in this study were raised in commercial conditions while most of the GWAS studies are performed in nucleus farms, which have more stringent biosecurity controls. The relevance of *Health* hits could therefore result from an *immunity* component embedded in the traits used in this study. The mediated effects colocalized with *Meat* QTL for most of the traits. This shows again the difference in the biological processes that the total and mediated effects are pointing at and, at least in this study, there was more consistency with previous literature by looking at the mediated than total effects. The G features that determine the P trait for the total effects are those associated with the trait (G → P). Conversely, the explicit modeling of the M−mediated path implies that a change in the G variable leads to a change in the M variables, in other words the genes associated with the G feature controls the gut microbiome composition (G → M). Once the latter is altered, i.e. a change in the abundance of the selected M features occurs, some physiological process leads to a change in fat deposition (M → P). The G features identified for the mediated effect would primarily affect the abundance of the mediator M feature. If such M mediator strongly impacts the fat deposition of the host, the mediated effect will be visible. Since some of the previously identified QTL for fat deposition in regions are in proximity to the G features identified for the mediated effect in this study, we could also hypothesize that the previous study captured genetic variation for the M mediator abundance, that was realized in the fat deposition phenotypes.

Table S4 reports the genes mapped to each of the top 15 windows for both the *total* and *mediated* effects on the two latent variables. Several genes identified withing the significant genomic regions for the mediated effects point at cognitive development. DYNLRB1 was found to be associated to neuronal survival [52], INPP5K was found to be associated to cognitive impairment [59], ZFHX2 was found to be associated to behavioral abnormalities [25] and CTNND2 was found to be associated to mental retardation and intellectual disability [31], [4]. Other genes were involved at cancer formation and development, for example AK4 was found to be involved in the esophageal cancer [62], RSG6 was found to be associated to bladder cancer [7], SLC43A2 was found to be involved in metastatic gastric cancer [61] and RPH3AL was found to be associated to colorectal adenocarcinoma [23]. As expected, other genes (PCK2, DOC2B) pointed at insulin regulation, diabetes and obesity [3], [42]. It should be kept in mind that these genes shape gut microbiome composition, before affecting fat deposition. Since a lot of genes and QTL seem to point at regulating health (in a broad sense) we could speculate that such regulation happens through the modification of the bacterial communities. The host genes will regulate the intestinal environment in order to favor certain species or fighting the insurgence of others. The bacterial species could be beneficial preventing the insurgence of tumors and regulating the gut-brain axis, for example.

### 4.4 Mediated effects as a new source of genetic variation

The G features with a significant mediated effect cannot be found in a traditional GWAS, unless they show a significant total effect, as would be the case in this study. This proves the value of a mediation analysis.

Mediation analysis has been used in other studies that aimed at estimating indirect effects that G could exert on P, even if using other host phenotypes rather than gut microbiome as a mediator. In a study on beef cattle performed with a different approach, Leal-Gutiérrez et al. [27] found different genomic regions affecting meat quality (expressed as a latent variable) either directly or mediated by carcass quality. Similarly, again with a different approach, Momen et al. [33] and Momen et al. [34] identified several genomic markers with phenotype-mediated effects in chicken and rice, respectively.

Therefore, the estimation of microbiome-mediated effects could help discover additional genetic variance for many traits. The role of gut microbiome has been previously proposed and as a partial explanation for the missing heritability problem [47], [13]. While most of the authors suggest using the microbiome-generated portion of phenotypic variance to the host genetic component in order to fill the missing heritability gap, here we suggest using mediated effects to fill such gaps. In our approach, the P phenotypic variance generated by the G → M → P path can be added to the regular G → P generated variance (heritability). The missing heritability gap could be filled by adding the G features involved in the mediated path could be added to the overall variance absorbed by the host genotype.

### 4.5 Pitfalls of the current study and future research

The current study was performed under commercial pork production conditions, and animals were fed standard diets. It is well known that the gut microbiome can be heavily influenced by feeding different diets or using pro-/pre-/anti-biotics. For this reason, the mediated effect should be studied in other populations or experiments because of its specificity. For example, let’s assume that the use of feed additives alters the gut microbiome composition, including a change in the M feature that is involved in the mediated path. We will call such external intervention as Z → M, where Z is some variable measuring the additive dosage. While the M → P effects would not be altered and would probably be observed on P, the G → M could be altered due to Z → M since the two effects are competing. If the external intervention ‘overwrites’ G → M, this effect could be nullified as Z is probably designed to totally control M. Alternatively, both effects could be magnified if some biological interaction between G and Z occurs, i.e. (GZ) → M. It is important to note that such susceptibility to external interventions could be found for the total effect as well, although the possibility for that effect to incorporate multiple paths makes it less prone to change under different conditions.

From a methodological standpoint, this study proposed a relatively simple method to test for mediations. Models that allow mediation through multiple variables should be studied. The discovery of genomic markers associated with (or affecting a) phenotype of interest should be performed with methods that can fit all markers within the same model, accounting for linkage disequilibrium and perhaps performing variable selection [12]. Some studies have proposed a correction of the phenotype of interest for the mediator [27], [60] and inferences are based on comparing the host genomic effects to a model that does not include the correction. To use the terminology developed in this study, such models would perform a comparison between the $\gamma_{p\leftarrow g}$ and ${\gamma \text{'}}_{p\leftarrow g}$ estimates, which is usually referred to as the Baron and Kenny approach [2]. While the method is correct, it doesn’t allow for the explicit estimation of the $\alpha_{m\leftarrow g}$ and its testing simultaneously to the other effects in the model [19].

The option to use more than one mediator variable should be allowed to fully describe the biological processes of mediation, especially if the proposed mediators show some degree of correlations. In this regard, Preacher and Hayes [40] proposed to use a multi-mediator model which could contrast the different paths, but this method would require the simultaneous estimation of multiple paths, which would in turn require large datasets. The potential use of latent variables as mediators has also been discussed, but the method may not be suitable for high-dimensionality data like microbiome profiles: a latent mediator built on several microbial features would exert a stronger M → P effect but there could be problems with the G → M effect. Due to the different genetic architecture of the several M features [5], the identification of which G feature affects which M feature is not trivial. The estimation of G effects on a latent M variable would imply the existence of multiple effects of G on the multiple M, which makes computation and interpretation difficult. In this regard, van Kesteren and Oberski [57] proposed a method for selection and regularization of the mediator variables taking into account correlations among mediators, which seems more suitable for the use on high-dimensional biological data.

## 5 Conclusions

In this study we propose a simple method to study the causal relationships between host genome, gut microbiome and host phenotypes. We used a dataset that included information collected on swine (*Sus scrofa*) where different measures of fat deposition served as phenotypes of interest.

This study shows that a large number of host genomic features affect these phenotypes through an indirect, empirically-tested, microbiome-mediated effect on measured and latent variables. It is possible biological that these genomic features contribute to controlling the composition of the gut microbiome, specifically affecting the abundance of certain taxa, which in turn can affect the rate of fat deposition. Many genomic features don’t affect the phenotype in a direct way and would not have been identified in a genome-wide association study performed without the inclusion of a microbiome mediator variable.

The example shown in this study suggests that some portion of the genetic variance for certain traits may not be evident when performing association studies. Considering the importance of understating the genetic architecture of certain traits, the implementation of a mediation analysis in structural equation modeling seems appealing. With the constant progress in generating high-dimensional biological data, the understanding of the interplay between the genes in the host and the genes of the microbes that live in the intestinal lumen is gaining more interest and this study proposes an approach to dissecting this relationship in a causal framework. In addition, the recovery of genetic (genomic) variance for certain traits could help solve the so called ‘missing heritability problem’. Future research should be aimed at studying the longitudinal causal network among the different variables as well as implementing models that allow the selection and regularization of exogenous and mediator variables.

## Author contributions

F.T. and C.M. conceived the study. F.T. performed the statistical analysis and wrote the first draft of the manuscript. C.M. contributed to the manuscript writing and the interpretation of results. J.F., Cl.S. and Ca.S. acquired the phenotypic data. All authors have read and approved the final manuscript.

## Declaration of Competing Interest

The authors declare that they have no known competing financial interests or personal relationships that could have appeared to influence the work reported in this paper.

## Figures and Tables

**Supplementary figure 1:**