# Supplementary material for: Gut microbiome mediates host genomic effects on phenotypes: a case study with fat deposition in pigs
Source: Comput Struct Biotechnol J. 2020 Dec 30;19:530–44. doi: 10.1016/j.csbj.2020.12.038 (PMC7809165; doi:10.1016/j.csbj.2020.12.038)
Supplement: Supplementary data 1 [file mmc1.pdf]

Table S1: Summary of results for the M1, M1L, M4 and M4L models.

Trait[1]	Total[2]		OTU[3]	Indirect[4]					
	P-val	Boot		Mediation	Sobel	Boot	Perm	Emp Test	Sobel & Emp
FATg	8,195	495	S2a	1069	96	181	0	0	0
			S2b	2311	339	464	812	464	209
BF1	6,168	237	S2a	1326	132	225	423	225	77
			S2b	2571	350	492	918	492	204
BF2	7,115	314	S2a	1255	140	230	434	230	95
			S2b	2337	321	461	793	461	196
BF3	7,890	273	S2a	1230	122	189	399	189	66
			S2b	1874	273	333	640	333	173
FATt	7,718	358	S2a	1240	142	238	451	238	86
			S2b	1611	174	279	552	279	108
			S3a	2971	525	722	0	0	0
			S3b	1420	138	258	2	1	0
			S3c	1707	237	344	616	344	149
BF4	8,440	482	S2a	1139	144	212	374	212	83
			S2b	1411	153	264	525	264	105
			S3a	2896	491	659	1,096	659	308
			S3b	1470	150	243	484	243	90
			S3c	1868	236	380	662	380	141
BFt	6,811	497	S2a	1136	110	202	422	202	76
			S2b	1580	187	299	569	299	119
			S3a	2383	360	526	907	526	230
			S3b	1217	96	207	382	207	63
			S3c	1607	212	292	533	292	136
BEL	5,906	265	S2a	1454	181	292	512	292	125
			S2b	1679	215	308	589	308	119
			S3a	3114	531	747	1,235	747	308
			S3b	1338	129	220	460	220	82
			S3c	1180	95	176	408	176	58

[1]Measured (host phenotypic) or latent variables used in the analysis.

[2]Number of host genomic features identified as significant for the total effect (G → P, models M1 or M1L). *P-val* reports the number of features showing *P*-value smaller than 0.05 for the Gamma effect. *Boot* reports the number of features resulting significant after bootstrapping.
[3]Gut microbial feature used as mediator for the estimation of the indirect effect (G → M → P, models M4 or M4L).
[4]Number of host genomic features identified as significant for the indirect effect (G → M → P, models M4 or M4L). *Mediation* reports the number of features showing a mediated effect larger than the direct effect. Moving to the right, the columns report the number of features in *Mediation* that were also significant for: Sobel test, Bootstrapping test, Permutation test, Bootstrapping and Permutation tests, all tests.

Table S2: Comparison of host genomic features found significantly affecting latent vs measured variables for the total effect (G → P, models M1 or M1L). The table reports the number of host genomic features identified as affecting latent variables which were also found affecting measured variables.

Latent variable[1]	Measured variable[2]	Total[3]
FATg	BF1	126
	BF2	249
	BF3	119
	None[6]	203
FATt	BF4	202
	BFt	216
	BEL	44
	None[6]	77

[1]Latent variables (host phenotypic) used in the analysis. FATg: subcutaneous backfat deposition from 118 to 174 days of life. FATf: backfat and belly deposition at slaughter (197 days).
[2]Measured variable (host phenotypic) used in the analysis. BF1, BF2, BF3 and BF4: measures of subcutaneous fat depth taken *in vivo* at the age of 118.1±1.16 d; 145.9±1.53 d, 174.3±1.43 d and 196.6±8.03 d, respectively. BFt: measure of subcutaneous fat depth taken *post mortem*. BEL: weight of belly cut taken *post mortem*.
[3]Number of host genomic features identified for the total effect (G → P, models M1 or M1L).

Table S3: Comparison of host genomic features found significantly affecting latent vs measured variables for the indirect effect (G → M → P, models M4 or M4L). The table reports the number of host genomic features identified as affecting latent variables which were also found affecting measured variables.

Latent variable[1]	OTU[2]	Measured variable[3]	Indirect[4]
FATg	S2a	BF1	0
		BF2	0
		BF3	0
		None[5]	0
	S2b	BF1	182
		BF2	276
		BF3	147
		None[5]	97
FATt	S2a	BF4	36
		BFt	82
		BEL	42
		None[5]	119
	S2b	BF4	56
		BFt	129
		BEL	52
		None[5]	109
	S3a	BF4	0
		BFt	0
		BEL	0
		None[5]	0
	S3b	BF4	1
		BFt	0
		BEL	0
		None[5]	0
	S3c	BF4	113
		BFt	116
		BEL	52
		None[5]	125

[1]Latent variables (host phenotypic) used in the analysis. FATg: subcutaneous backfat deposition from 118 to 174 days of life. FATf: backfat and belly deposition at slaughter (197 days).

[2]Gut microbial feature used as mediator for the estimation of the indirect effect (G → M → P, models M4 or M4L).

[3]Measured variable (host phenotypic) used in the analysis. BF1, BF2, BF3 and BF4: measures of subcutaneous fat depth taken *in vivo* at the age of 118.1±1.16 d; 145.9±1.53 d, 174.3±1.43 d and 196.6±8.03 d, respectively. BFt: measure of subcutaneous fat depth taken *post mortem*. BEL: weight of belly cut taken *post mortem*.

[4]Number of host genomic features identified for the indirect effect (G → M → P).

[5]Indicates the number of host genomic features that were not identified using the measured variables.

Table S4: Top 15 host genomic regions with significant mediated or total effect on the latent variables. Effects on FATg were mediated by S2b (*Peptococcus*) and effects on FATt were mediated by S3c (*Butyricicoccus*).

Trait	Effect	Rank	Chromosome	Start position (bp)	End position (bp)	Effect (abs.)	Genes
FATg	Mediated	1	17	37920822	37938790	0.0162	DYNLRB1
		2	7	134388813	134388813	0.0161	
		3	2	30870402	30999341	0.0160	
		4	10	66364425	66364425	0.0146	
		5	6	147237661	147237661	0.0146	AK4
		6	16	7358991	7358991	0.0146	
		7	16	9664975	9811863	0.0145	
		8	7	91966976	91966976	0.0144	RAD51B
		9	16	2931783	2931783	0.0143	
		10	7	95549575	95549575	0.0143	RGS6
		11	7	75121635	75830988	0.0142	REC8, PSME2, EMC9, PCK2, NRL, CPNE6, CARMIL3, JPH4, AP1G2, THTPA, ZFHX2, MIR208A, IL25, EFS, SLC22A17, PPP1R3E, HOMEZ, RNF212B
		12	5	110792250	110792250	0.0141	
		13	4	12603508	12603508	0.0137	
		14	8	134362111	134362111	0.0136	
		15	13	205628626	205687638	0.0136	ABCG1, TFF3, TFF2
FATg	Total	1	3	126974179	126974179	0.1013	ASAP2
		2	5	28604085	28604085	0.0997	SRGAP1, C12orf66
		3	2	59957369	60318677	0.0988	FCHO1, MAP1S, COLGALT1, PGLS, SLC27A1,

						NXNL1, MVB12A, CCDC194
	4	1	219816097	221276670	0.0956	SMARCA2, DMRT2, DMRT3, KANK1, DOCK8
	5	1	193610978	194258004	0.0950	ESR2, SYNE2
	6	9	8014440	8014440	0.0923	FAM168A
	7	7	4076029	4095479	0.0922	
	8	1	74467285	75058189	0.0920	AFG1L, ARMC2, SESN1
	9	15	118809721	118809721	0.0900	
	10	1	183336668	185654681	0.0887	BMP4, CDKN3, GMFB, CGRRF1, SAMD4A, GCH1, WDHD1, SOCS4, MAPK1IP1L, LGALS3, DLGAP5, FBXO34, ATG14, TBPL2, KTN1, PELI2
	11	13	95481059	99700583	0.0881	PLCH1, GMPS, KCB1, TIPARP, CCNL1, VEPH1, SHOX2, RSRC1, GFM1, LXN, RARRES1, MFSD1, IQCJ
	12	6	63310472	63895672	0.0872	SAMD11, NOC2L,

							KLHL17, PLEKHN1, HES4, AGRN, RNF223, TNFRSF4, SDF4, B3GALT6, C1QTNF12, UBE2J2, ACAP3, PUSL1, INTS11, DVL1, MXRA8, AURKAIP1, CCNL2, MRPL20, VWA1, ATAD3A, TMEM240, MIB2, MMP23B, SLC35E2B, GNB1
		13	18	9598458	9979869	0.0870	KDM7A, PARP12, TBXAS1, HIPK2
		14	13	77535708	77558751	0.0869	STAG1
		15	1	189896026	189896026	0.0867	MT1
FATt	Mediated	1	9	26759246	26759246	0.0143	
		2	5	63702753	63702753	0.0134	
		3	7	65904152	65904152	0.0128	
		4	4	34938520	34938520	0.0124	NCALD
		5	12	47396819	47809068	0.0122	RPH3AL, DOC2B, CRK, MYO1C, INPP5K, PITP, SLC43A2
		6	16	958609	958609	0.0121	CTNND2
		7	7	62371545	62438211	0.0118	TTC6
		8	14	26641666	26641666	0.0117	
		9	7	90289029	90329629	0.0113	CCDC196

		10	1	33781901	33781901	0.0112	
		11	2	5888217	5888217	0.0112	
		12	7	45236888	45394771	0.0112	
		13	2	7272254	7272254	0.0111	ATG2A
		14	7	27550218	27550218	0.0110	KHDRBS2
		15	3	129063098	129063098	0.0109	
FATt	Total	1	1	242984908	242984908	0.0918	PLPPR1
		2	2	59957369	60261433	0.0910	FCHO1, MAP1S, COLGALT1, PGLS, SLC27A1, NXNL1
		3	5	28604085	28604085	0.0910	SRGAP1, C12orf66
		4	11	20953383	20953383	0.0864	
		5	3	103288341	103288341	0.0853	STRN
		6	5	68326348	68505852	0.0834	ERC1
		7	1	193610978	194258004	0.0820	ESR2, SYNE2
		8	3	83365231	87200902	0.0810	FANCL, VRK2, CCDC85A, EFEMP1, PNPT1, CCDC88A, MTIF2, RPS27A, CLHC1, RTN4, EML6, SPTBN1, C2orf73
		9	14	1000181	1000181	0.0804	SEMA4D
		10	3	135861845	135861845	0.0803	
		11	1	240661253	241120799	0.0802	COL15A1, TGFBR1, ALG2
		12	18	9598458	9979869	0.0799	KDM7A, PARP12, TBXAS1, HIPK2
		13	2	132702219	132702219	0.0797	CHSY3
		14	1	284208765	284208765	0.0795	
		15	11	68288395	68312256	0.0791	UBAC2
